# Supplementary material for: The translocation of a chloride channel from the Golgi to the plasma membrane helps plants adapt to salt stress
Source: Nat Commun. 2024 May 10;15:3978. doi: 10.1038/s41467-024-48234-z (PMC11087495; doi:10.1038/s41467-024-48234-z)
Supplement: Supplementary file 1 — Supplementary Info [file 41467_2024_48234_MOESM1_ESM.pdf]

Online Supplementary information for

The translocation of a chloride channel from the Golgi to the plasma membrane helps plants adapt to salt stress

Sivamathini Rajappa[1**], Pannaga Krishnamurthy[1,2**], Hua Huang[3,4,5,6], Dejie Yu[3,4,5,6], Jiří Friml[7], Jian Xu[8], and Prakash P Kumar[1,2*]

[1] Department of Biological Sciences and Research Centre on Sustainable Urban Farming, National University of Singapore, 14 Science Drive 4, Singapore 117543

[2] NUS Environmental Research Institute, National University of Singapore, #02-01, T-Lab Building, 5A Engineering Drive 1, Singapore 117411

[3] Department of Physiology, Yong Loo Lin School of Medicine, National University of Singapore, Singapore 117597

[4] Electrophysiology Core Facility, Yong Loo Lin School of Medicine, National University of Singapore, Singapore 117456

[5] Healthy Longevity Translational Research Program, Yong Loo Lin School of Medicine, National University of Singapore: Level 5, Centre for Life Sciences, 28 Medical Drive, Singapore 117456

[6] Cardiovascular Diseases Program, National University of Singapore, 14 Medical Drive, MD6, #08-01, Singapore 117599

[7] Institute of Science and Technology Austria (IST Austria) Am Campus 1, 3400 Klosterneuburg, Austria (jiri.friml@ist.ac.at)

[8] Department of Plant Systems Physiology, Radboud Institute for Biological and Environmental Sciences, Radboud University, Huygens Building, Heyendaalseweg 135, 6500 AJ Nijmegen, The Netherlands (J.Xu@science.ru.nl)

[**]These authors contributed equally

[*]Corresponding author

Address: Department of Biological Sciences, National University of Singapore, 14 Science Drive 4, Singapore 117543

Tel: +65-65162859

Fax: +65-67792486

email: prakash.kumar@nus.edu.sg

Methods

Plasma membrane (PM) isolation by two-phase partitioning and formation of Inside-Out Vesicles

Arabidopsis seedlings (150 g FW) that were subjected to 6 hours of 100 mM NaCl treatment were sliced in small pieces before homogenizing in a blender with 100 ml of ice-cold homogenization buffer [50 mM Tris base, 500 mM sucrose, 10 % (w/v) glycerol, 20 mM EDTA, 20 mM EGTA, 50 mM NaF, 5 mM β-glycerophosphate, 1 mM phenantroline, 0.6 % (w/v) PVP, and 10 mM ascorbic acid][1]. This was then filtered through a 100 µm nylon mesh and the filtrate was centrifuged at 10,000 ×g for 30 min, at 4 °C. Microsomal membranes were obtained by centrifuging the supernatant at 84000 ×g for 30 min at 4 °C and later resuspended in 9 ml of microsomal buffer (5 mM phosphate buffer pH 7.8, 330 mM sucrose, 2 mM DTT and 10 mM NaF)[1]. Plasma membrane vesicles were later isolated using two-phase partitioning by adding microsomal membranes onto a 27 g PEG-dextran mixture with a final composition of PEG-3350/Dextran-T500 6.4% (w/w), in the presence of 5 mM KCl, 0.3 M sucrose and 5 mM potassium phosphate buffer (pH 7.8). This mixture was centrifuged for 5 min at 4000 ×g. The upper phase was transferred to another tube, washed with a solution containing 5 mM phosphate buffer and 0.3 M sucrose, pH 7.8, and centrifuged at 176,000 ×g for 1 hour at 4 °C in a Beckman Coulter ultracentrifuge. Pelleted PM vesicles were resuspended in PM washing buffer (10 mM Tris base, 10 mM boric acid, 300 mM sucrose, 9 mM KCl, 5 mM EDTA, 5 mM EGTA and 50 mM NaF) supplemented with protease inhibitor and 5 mM DTT, subsequently frozen in liquid nitrogen and stored at -80 °C for future use. This mainly consisted of the right-side-out vesicles. A portion of these vesicles were turned inside-out by carrying out 8 rounds of freeze/thaw cycles[2]. The sealed inside-out and right-side-out vesicles were subsequently separated by repeating the phase partition step described above. Increasing the number of freeze/thaw cycles significantly increased the yield of inside-out vesicles. These vesicles were loaded with a fluorophore (MQAE, which is quenched by Cl^- ions) and used to check the transmembrane Cl^- transport activity (see Supplementary Fig. 5).

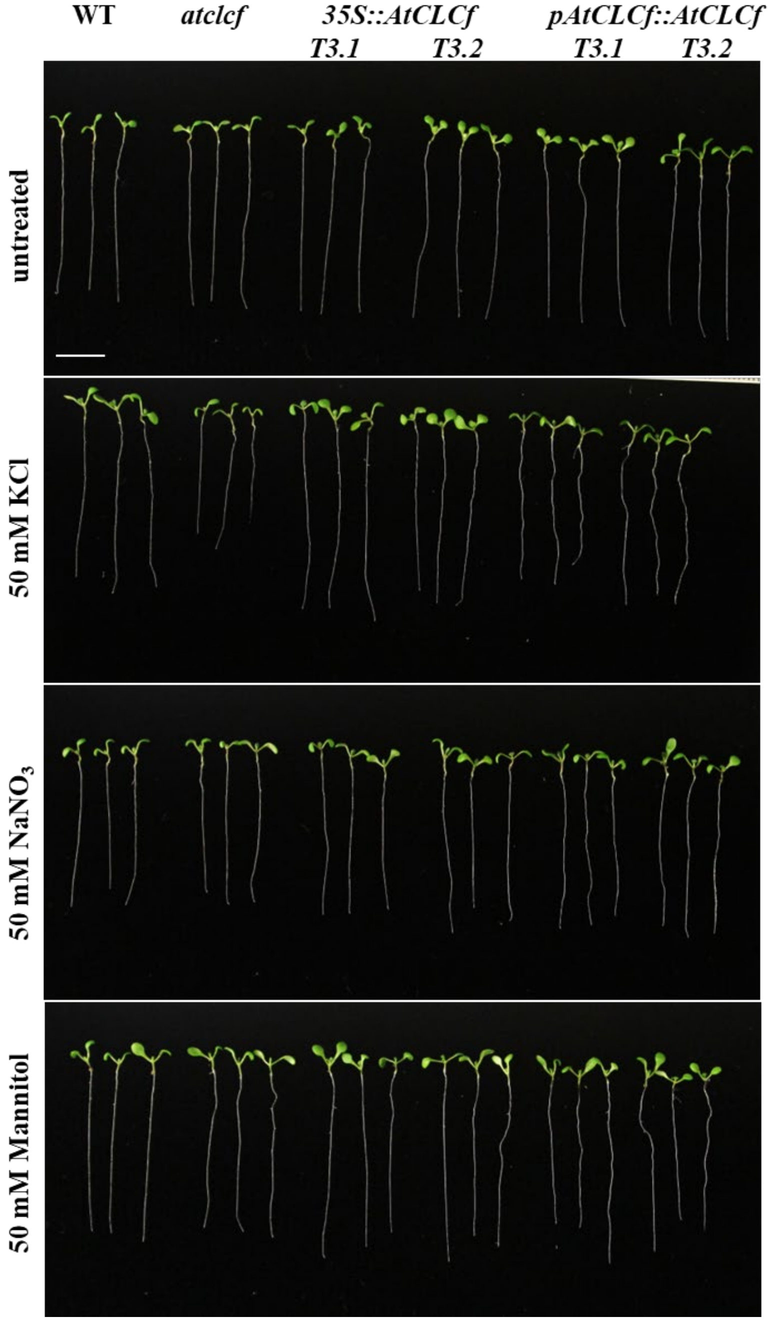

Supplementary Fig. 1: **KCl affects the growth of *atclcf* mutants but not $NaNO_3$ and mannitol.** Arabidopsis WT, a*tclcf*, *35S::AtCLCf;atclcf* and *pAtCLCf::AtCLCf;atclcf* plants were grown on MS agar with and without 50 mM KCl, 50 mM $NaNO_3$ or 50 mM mannitol treatment. The images were taken at 7 days after germination, scale bar = 1 cm. Data were obtained from three independent experiments, and representative images are shown here.

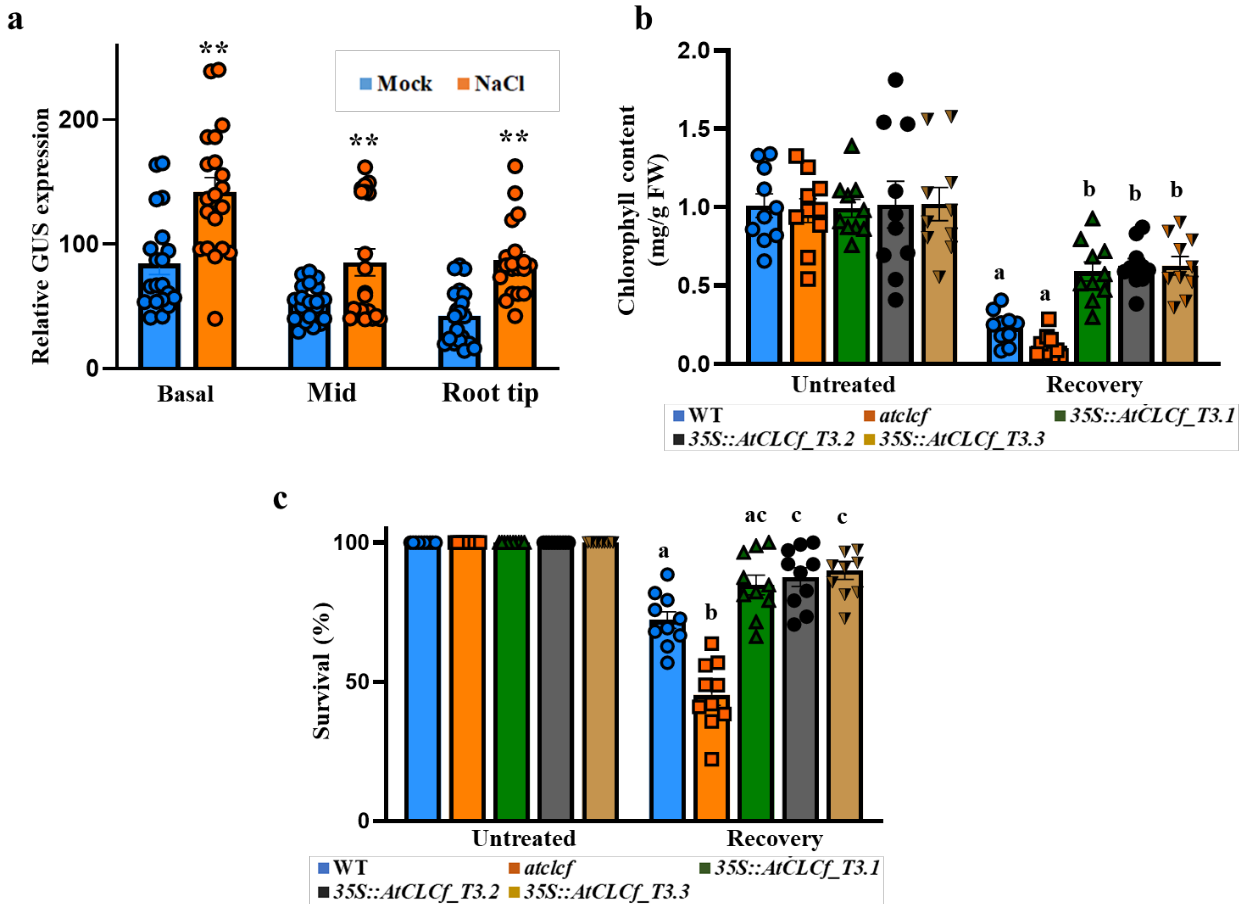

Supplementary Fig. 2: **Ectopic expression of *AtCLCf* enhances salt tolerance in Arabidopsis.** **(a)** Relative quantification of GUS intensity before (mock) and after NaCl treatment. Data are mean ± SE, $n = 20$. Asterisks indicate statistically significant differences (* = $P < 0.05$, ** = $P < 0.01$) as measured by unpaired Student's *t*-test (two-tailed) between mock and treatment. **(b)** Chlorophyll contents, data are mean ± SE ($n = 10$). **(c)** survival rates of WT, *atclcf*, and *35S::AtCLCf* plants before and after recovery from salt treatment, data are mean ± SE ($n = 10$). Means with different letters within a data set are significantly different, $P > 0.05$ (One-way ANOVA followed by Tukey's test).

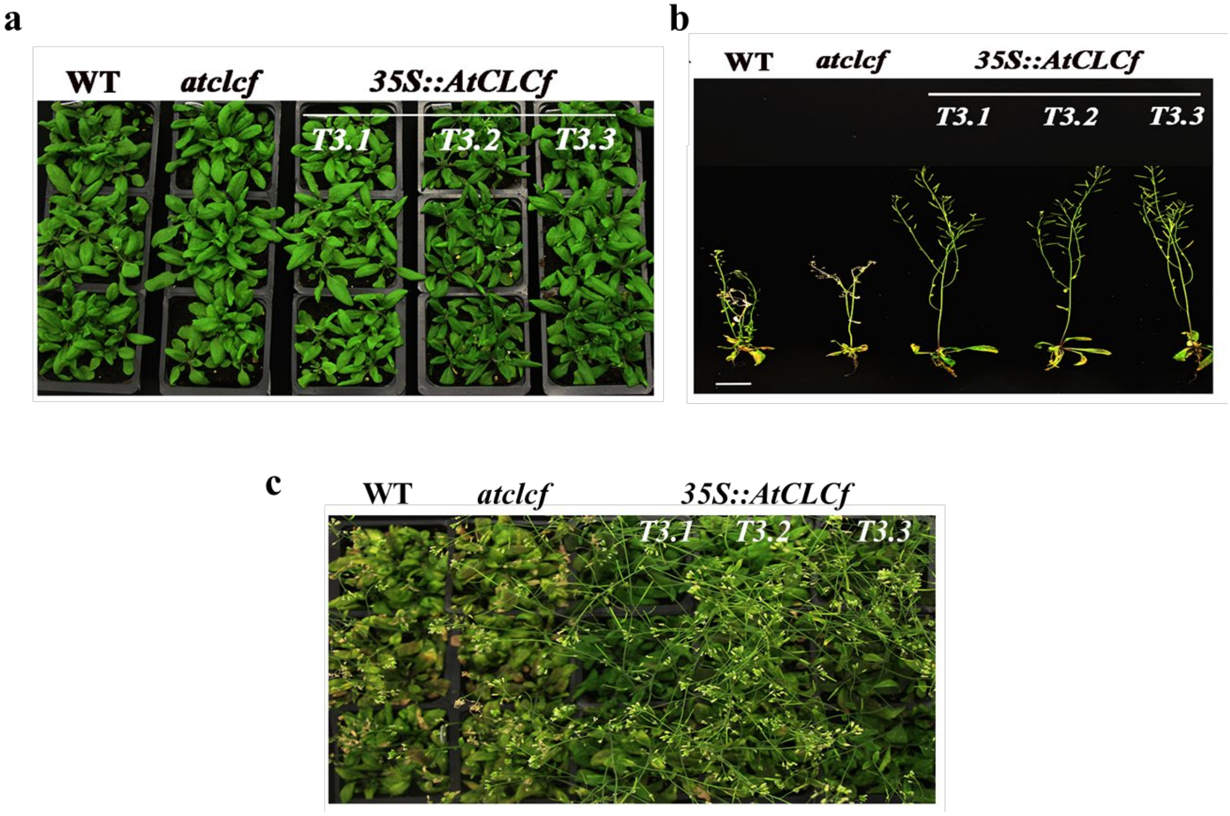

Supplementary Fig. 3: **Ectopic expression of *AtCLCf* enhances salt tolerance in Arabidopsis.** (**a)** Growth response of one-month-old WT, *atclcf* and *35S::AtCLCf* lines grown in soil without and (**b**, **c**) with 150 mM NaCl treatment for one week, followed by recovery growth in normal water (without NaCl) for one more week, scale bar = 30 mm. Data were obtained from three independent experiments, and representative images are shown here.

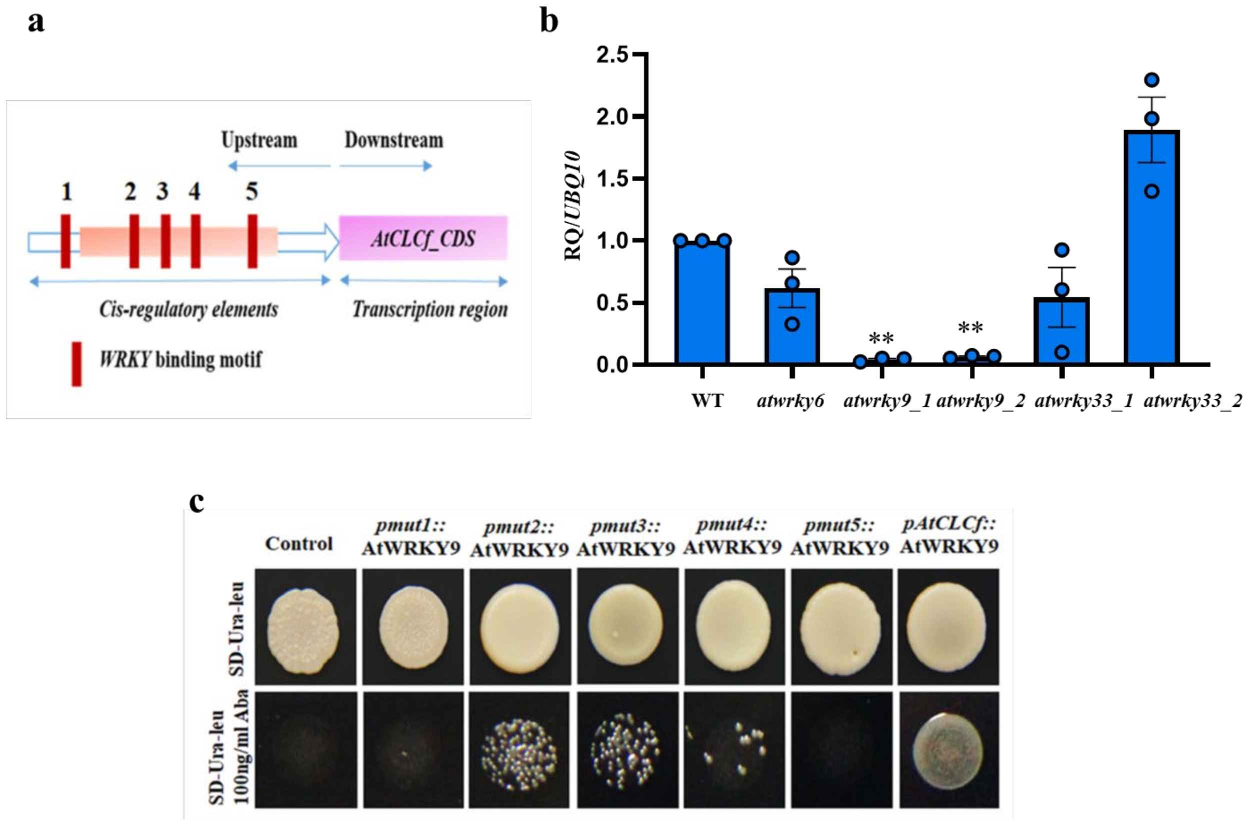

Supplementary Fig. 4: **AtWRKY9 transcription factor acts as an upstream regulator of *AtCLCf*.** (**a**) Schematic representation of the presence of binding motifs for WRKY transcription factors in the upstream region of *AtCLCf*. (**b**) Transcript levels of *AtCLCf* in the *atwrky6*, *atwrky9* and *atwrky33* T-DNA insertional mutants compared to the wild type (WT). Data are mean ± SE, (n = 3, with 3 biological replicates, each with 3 technical replicates). Asterisks indicate statistically significant differences (** = $P < 0.01$) as measured by unpaired Student's t-test (two-tailed) between WT and mutants. (**c**) Yeast one hybrid (Y1H) assay shows regulation of *AtCLCf* by AtWRKY9. Mutated WRKY9 binding domains (pm1, pm2, pm3, pm4 and pm5 *AtCLCf*) were used as additional controls. The representative growth status of yeast cells is shown on SD/-Leu agar medium without and with 100 ng of aureobasidin A. Y1H data were obtained from three independent experiments.

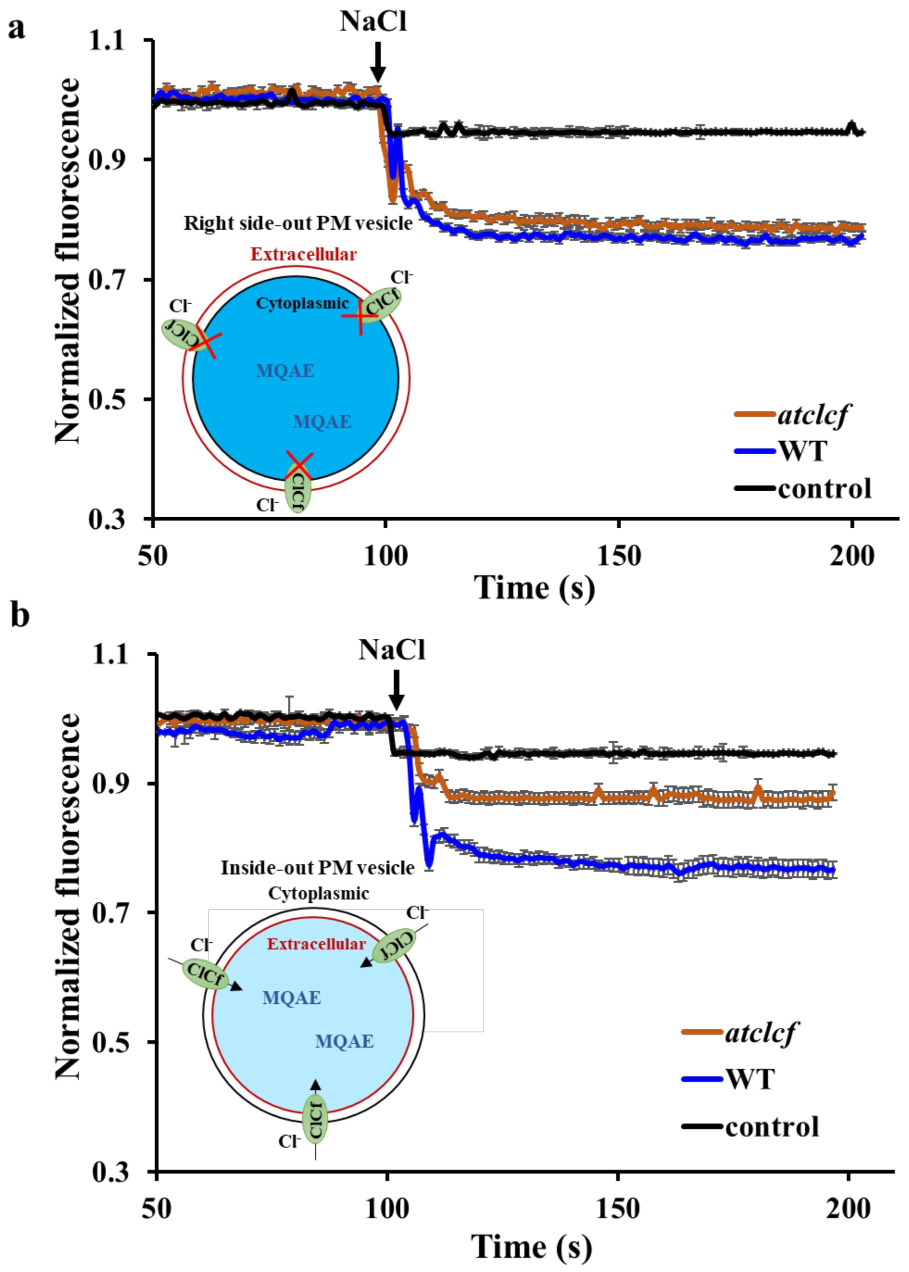

Supplementary Fig. 5: **Cl^- transport in PM vesicles.** Results of Cl^- transport assay in control (only buffer added without NaCl) and after addition of 50 mM NaCl to PM vesicles loaded with 5 mM MQAE fluorophore. PM vesicles were prepared from WT and *atclcf* Arabidopsis plants. **(a)** No quenching of fluorescence was seen in the right-side-out (RSO) PM vesicles (dark blue vesicle, inset; *n* = 3). **(b)** Quenching of fluorescence was seen in the inside-out (ISO) PM vesicles (light blue vesicle, inset; *n* = 3). Data in a and b are mean ± SD. Insets were created using PowerPoint software.

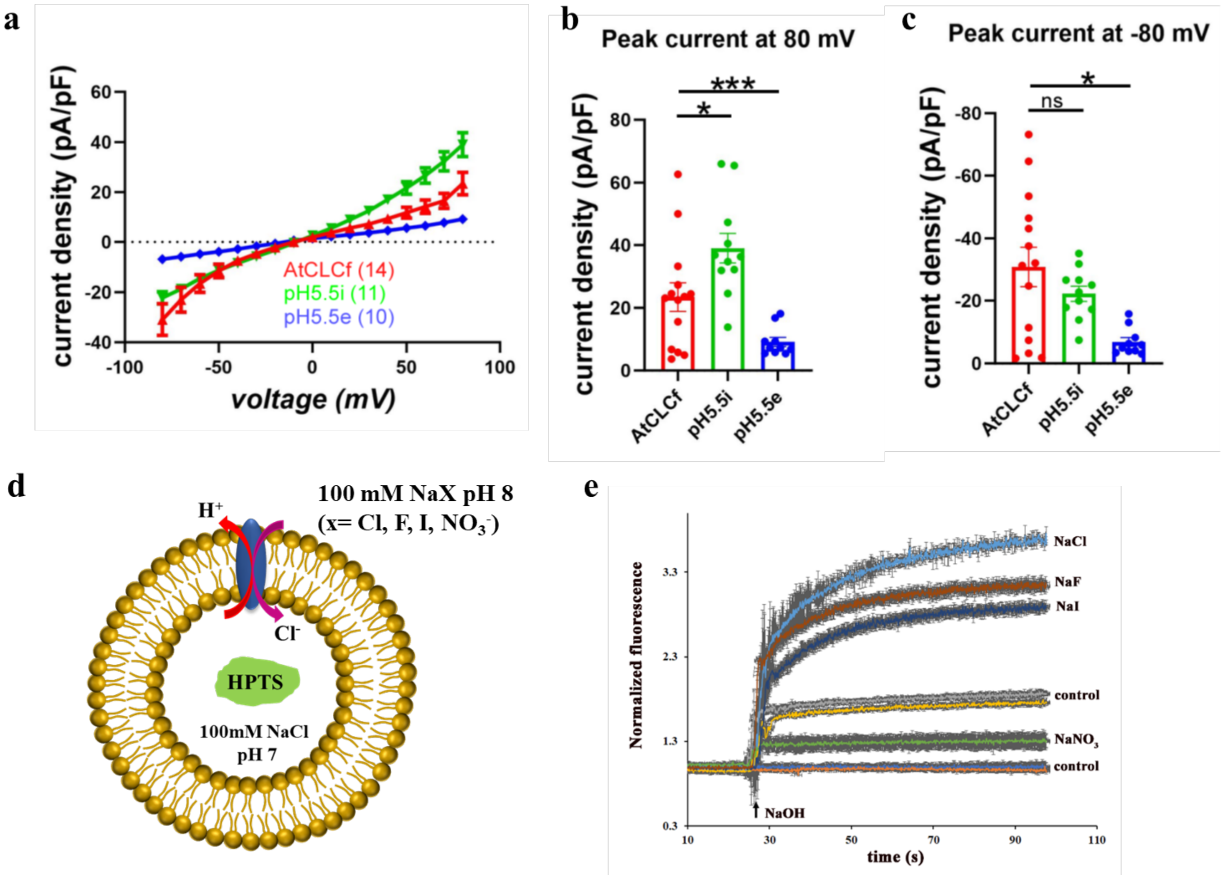

Supplementary Fig. 6: **AtCLCf functions as a Cl^-/H^+ antiporter.** Electrophysiological **(a-c)** and liposome-based **(d-e)** assays were performed to check the effect of pH on the transport properties of AtCLCf. (**a**) Current voltage relationship of AtCLCf transfected Human Embryonic Kidney 293 (HEK293FT) cells recorded with internal and external solution at pH 7.4 as compared to cells recorded with internal solution at pH 5.5 (pH 5.5i) and external solution at pH 5.5 (pH 5.5e), Data are mean ± SE n = (14 at pH 7.4, 11 at pH 5.5i, 10 at pH 5.5e). (**b**) and (**c**) data comparing the maximal outward and inward current at 80 and -80 mV, respectively. * $p < 0.05$, *** $p < 0.001$, ns, non-significant as indicated by unpaired Student's t-test (two-tailed). The averaged current density was obtained by normalizing the peak current over the cell capacitance of individual cells prior to averaging. (**d**) Schematic representation of HPTS-based fluorescence assay under pH gradient (inside pH 7 and outside pH 8). The liposome image was created using PowerPoint software. (**e**) HPTS-based fluorescence assay to check of addition of NaCl, NaF and NaI and $NaNO_3$ on transport of H^+ ions by AtCLCf. Liposomes without AtCLCf incorporation were used as controls. Data are mean ± SD, n = 3.

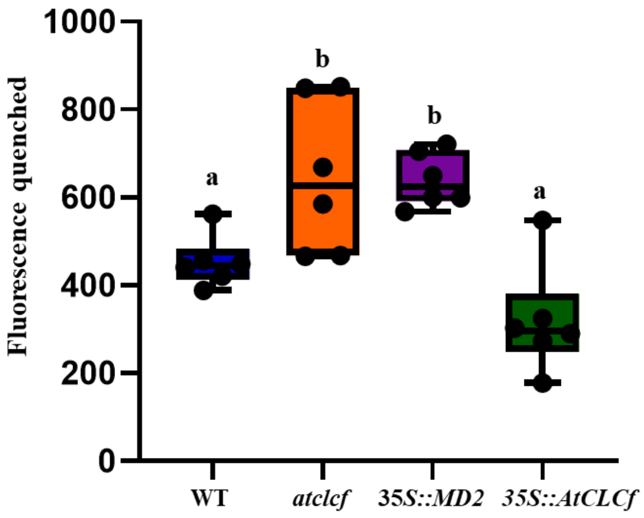

Supplementary Fig. 7: Quenching of fluorescence (a measure of Cl^- ion concentration) was measured from the protoplasts treated with 50 mM NaCl for 3 h followed by 100 µM MQAE loading for 30 min, $n = 6$. Data are mean ± SE. Means with different letters are significantly different, $P < 0.05$ (one-way ANOVA followed by Tukey's test).

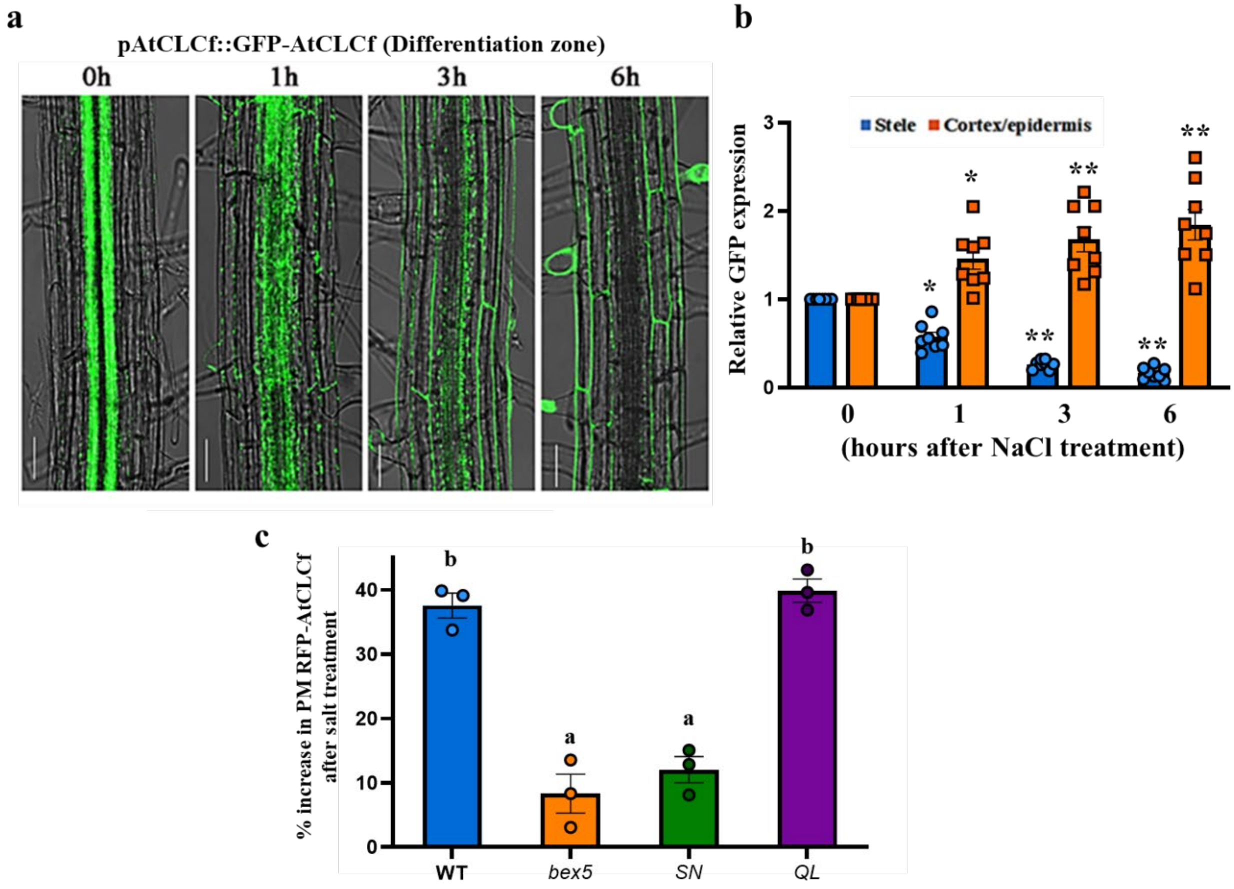

Supplementary Fig. 8: **Effect of NaCl on the localization of AtCLCf. (a)** GFP fluorescence in the root differentiation zone of plants expressing *pAtCLCf::GFP-AtCLCf* with and without NaCl treatment. Images were captured from three independent experiments, and representative images are shown. **(b)** Quantification of GFP expression in (a) Data are mean ± SE, $n = 8$. Asterisks in (b) indicate statistically significant differences (* = $P < 0.05$, ** = $P < 0.01$) as measured by unpaired Student's *t*-test (two-tailed) between untreated and the treated. The GFP signal was visualized at 488 nm excitation, 500-525 nm emission Scale bar =15 µm. **(c)** Number of WT, *bex5*, *SN and QL* leaf protoplasts were scored for RFP-AtCLCf expression in the Golgi apparatus and PM. The percentage of protoplasts showing PM localization in salt treated and untreated conditions are shown. Data are mean ± SE, $n = 20$. Means with different letters are significantly different, $P > 0.05$ (One-way ANOVA followed by Tukey's test).

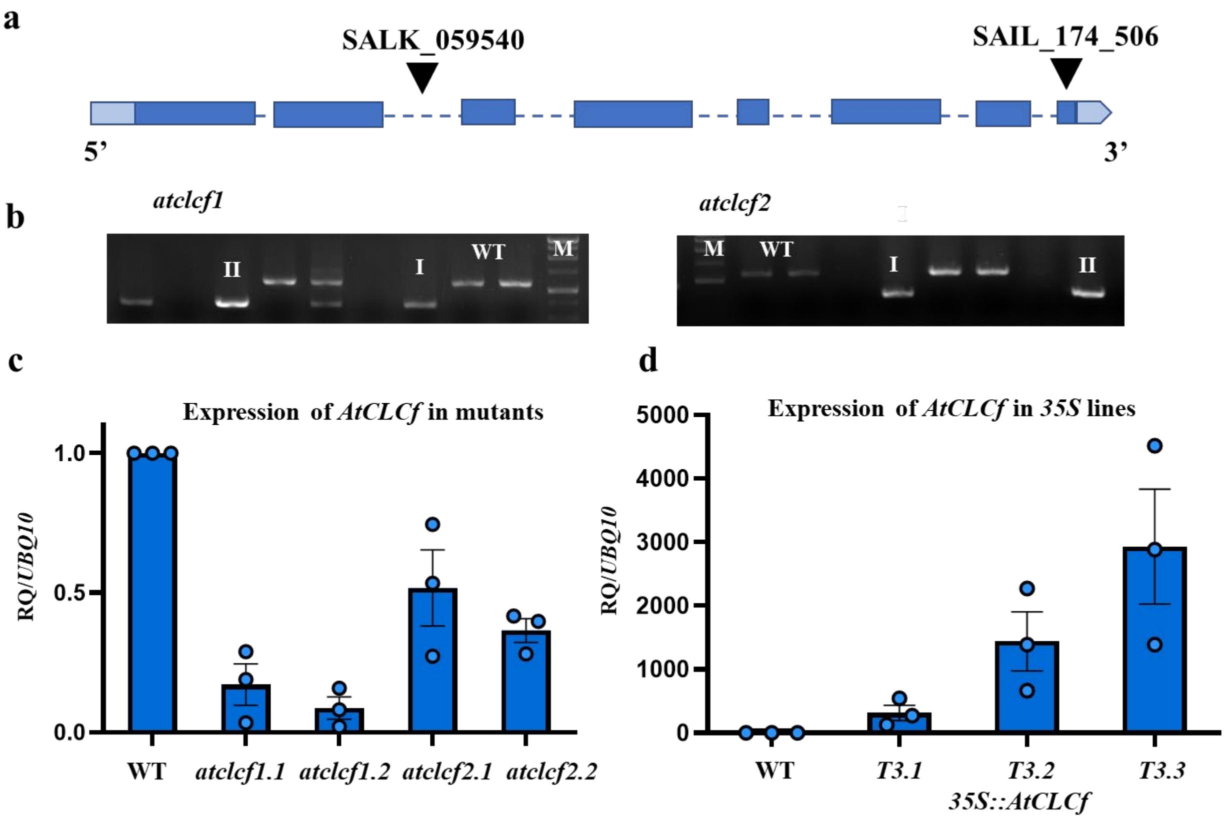

Supplementary Fig. 9: **Details about the *atclcf* T-DNA insertion mutants and qRT-PCR analysis of mutants and ectopic expression lines.** (**a**) Genetic map of *AtCLCf* T-DNA insertion. (**b**) Genotyping of *atclcf* shows the homozygous T-DNA insertion. Genotyping was carried out once. 1 Kb DNA ladder (1st base, Singapore) was used as marker. **(c)** qRT-PCR shows reduced expression of *AtCLCf* in *atclcf* T-DNA insertional mutants. (**d**) qRT-PCR shows increased expression of *AtCLCf* in ectopic expression lines. Relative expression levels of transcripts with reference to *AtUbiquitin 10* transcript levels are plotted. Data are mean ± SE, n = 3 (3 biological replicates, each with 3 technical replicates).

```
Optimized 1   ATGTCTTCTGGTGGTGCTGGTGAATACAACGAGGATAGACATTTGTTGAGATCTACTGAT
Original 1    ATGTCGTCAGGTGGGGCAGGGGAATACAATGAAGACCGCCATCTCCTGAGGAGTACCGAC
Optimized 61  GGAGATGAAGTTGGTATTGGTGGTGGTGAAGGAGATTTGGATGTTGAGTCTCAATCTCCA
Original 61   GGAGACGAGGTTGGGATAGGCGGGGGTGAAGGAGACCTTGATGTGGAAAGCCAGTCACCC
Optimized 121 GCTATTAGATCTGGTGCTGGTGGTGTTAGAGATTTGTTTAAGCACATCGATAGAAGATTC
Original 121  GCTATAAGGTCGGGCGCCGGCGGGGTCCGGGACCTATTCAAACATATTGACCGCCGTTTC
Optimized 181 TCTTTGTCTGGTAGAAGATTGTCTTTTAAAAGAATGGAAAACATTAGAGTTGATAGAGAG
Original 181  TCATTATCTGGTCGTCGGTTGAGTTTCAAGCGTATGGAGAATATCCGTGTGGATAGGGAG
Optimized 241 AGACATAATCCTTCTTCTTCTTCTGCTTTCTCTGCTGCTGGTGAAGAGGATGGTGGTGGT
Original 241  CGCCACAATCCGTCTTCTAGTTCAGCATTTAGCGCGGCCGGAGAGGAGGACGGGGGCGGA
Optimized 301 ATTTCTAACTTGCACTCTGTTGATGATAGAAACGATGAATACGGTTTCGATGAAGAGGTT
Original 301  ATATCTAATTTACACTCAGTGGACGATCGTAACGATGAATATGGGTTTGACGAAGAGGTT
Optimized 361 TTGGGAGATTCTGCTCCACCTGAGTGGGCTTTGTTGTTGATTGGTTGTTTGATTGGTGTT
Original 361  CTGGGTGACTCTGCTCCACCGGAGTGGGCACTACTATTGATCGGATGTCTCATTGGCGTA
Optimized 421 GCTGCTGGTATTTGTGTTGCTGGTTTTAACAAGGGTGTTCATGTTATTCACGAATGGGCT
Original 421  GCTGCGGGCATCTGCGTCGCAGGCTTCAACAAAGGGGTGCACGTGATACACGAATGGGCG
Optimized 481 TGGGCTGGTACTCCAAATGAGGGTGCTGCTTGGTTGAGATTGCAAAGATTGGCTGATACT
Original 481  TGGGCGGGTACACCTAATGAGGGTGCAGCTTGGCTCCGGCTGCAAAGACTTGCGGATACA
Optimized 541 TGGCATAGAATTTTGTTGATTCCTGTTACTGGTGGTGTTATTGTTGGTATGATGCACGGT
Original 541  TGGCATCGAATTCTGTTAATTCCGGTCACGGGGGGGGTTATTGTGGGCATGATGCATGGT
Optimized 601 TTGTTGGAAATCTTGGATCAAATCAGACAATCTAACTCTTCTCAAAGACAAGGTTTGGAT
Original 601  TTGTTAGAGATCTTAGACCAAATCCGACAGAGTAACTCTTCTCAGCGGCAGGGGCTGGAC
Optimized 661 TTCTTGGCTGGTATCTACCCAGTTATTAAGGCTATTCAAGCTGCTGTTACTTTGGGTACT
Original 661  TTTCTAGCTGGCATATATCCTGTAATTAAAGCCATCCAAGCTGCCGTTACTCTTGGCACG
Optimized 721 GGTTGTTCTTTGGGTCCAGAGGGTCCTTCTGTTGATATTGGTAAATCTTGTGCTAATGGT
Original 721  GGGTGTTCATTAGGTCCCGAGGGCCCCTCTGTCGACATAGGGAAGTCATGCGCGAACGGC
Optimized 781 TTTGCTTTGATGATGGAAAACAATAGAGAGAGAAGAATTGCTTTGACTGCTGCTGGTGCT
Original 781  TTTGCACTGATGATGGAAAACAATCGGGAGAGAAGGATCGCTCTCACGGCCGCGGGCGCC
Optimized 841 GCTTCTGGTATTGCTTCTGGTTTTAACGCTGCTGTTGCTGGTTGTTTCTTTGCTATTGAA
Original 841  GCATCCGGAATTGCATCCGGTTTCAACGCAGCAGTAGCTGGATGCTTTTTTGCTATAGAA
```

```
Optimized 901  ACTGTTTTGAGACCTTTGAGAGCTGAAAACTCTCCACCATTCACTACTGCTATGATCATC
Original  901  ACCGTCTTGAGACCATTGCGAGCGGAAAACAGCCCACCGTTTACGACCGCGATGATTATA
Optimized 961  TTGGCTTCTGTTATTTCTTCTACTGTTTCTAACGCTTTGTTGGGTACTCAATCTGCTTTT
Original  961  CTAGCCTCCGTGATCTCATCCACAGTGTCGAATGCACTTTTGGGAACACAATCCGCGTTT
Optimized 1021 ACTGTTCCATCTTACGATTTGAAGTCTGCTGCTGAATTGCCTTTGTATTTGATTTTGGGA
Original  1021 ACCGTGCCATCGTATGACCTAAAATCCGCTGCCGAGCTCCCCCTCTACCTAATCCTGGGT
Optimized 1081 ATGTTGTGTGGTGCTGTTTCTGTTGTTTTCTCTAGATTGGTTACTTGGTTCACTAAGTCT
Original  1081 ATGCTGTGCGGTGCTGTCAGTGTAGTGTTTTCGAGATTAGTGACATGGTTCACTAAATCC
Optimized 1141 TTCGATTTCATCAAGGATAAGTTCGGTTTGCCAGCTATTGTTTGTCCTGCTTTGGGTGGT
Original  1141 TTTGATTTCATTAAGGACAAGTTCGGGTTGCCAGCAATCGTTTGTCCCGCCCTCGGAGGT
Optimized 1201 TTGGGTGCTGGTATTATTGCTTTGAAGTACCCAGGTATTTTGTATTGGGGTTTTACTAAC
Original  1201 TTAGGCGCCGGTATAATTGCCCTCAAATATCCTGGTATACTGTATTGGGGATTTACTAAT
Optimized 1261 GTTGAAGAGATTTTGCATACTGGTAAATCTGCTTCTGCTCCTGGTATTTGGTTGTTGGCT
Original  1261 GTGGAAGAAATATTACACACAGGTAAAAGCGCATCCGCGCCAGGTATTTGGCTGCTGGCT
Optimized 1321 CAATTGGCTGCTGCTAAGGTTGTTGCTACTGCTTTGTGTAAAGGTTCTGGTTTGGTTGGT
Original  1321 CAGTTAGCCGCGGCAAAGGTAGTTGCGACCGCCCTTTGTAAGGGAAGCGGACTAGTAGGT
Optimized 1381 GGTTTGTACGCTCCATCTTTGATGATTGGTGCTGCTGTTGGTGCTGTTTTCGGTGGTTCT
Original  1381 GGATTGTACGCCCCCAGCCTAATGATTGGCGCTGCTGTGGGTGCTGTTTTCGGGGGCTCG
Optimized 1441 GCTGCTGAAATTATTAACAGAGCTATTCCTGGTAATGCTGCTGTTGCTCAACCTCAAGCT
Original  1441 GCCGCAGAGATAATAAACAGGGCAATCCCTGGTAATGCCGCTGTCGCTCAGCCGCAGGCG
Optimized 1501 TACGCTTTGGTTGGTATGGCTGCTACTTTGGCTTCTATGTGTTCTGTTCCTTTGACTTCT
Original  1501 TACGCGTTGGTCGGCATGGCGGCGACATTGGCTTCTATGTGTAGCGTCCCTCTCACAAGC
Optimized 1561 GTTTTGTTGTTGTTCGAGTTGACTAAGGATTACAGAATTTTGTTGCCATTGATGGGTGCT
Original  1561 GTATTACTATTATTCGAGCTCACGAAAGATTATAGGATCTTGTTGCCACTCATGGGAGCC
Optimized 1621 GTTGGTTTGGCTATTTGGGTTCCTTCTGTTGCTAATCAGGGTAAAGAATCTGATTCTTCT
Original  1621 GTTGGTTTAGCCATCTGGGTCCCGAGTGTAGCAAATCAAGGAAAGGAGTCGGATTCGAGT
```

```
Optimized 1681 GAGGGTAGATCTACTGGTAGAGGTTATTCTTCTTTGTCTCCATCTGAAAGAAAAACTGAG
Original  1681 GAAGGGCGAAGTACTGGCCGGGGATACTCGAGTCTGTCGCCTAGTGAGCGTAAAACGGAG
Optimized 1741 GGTGTTTGGAGACATACTGATAACGCTGATTCTTTGGAATTGACTGTTATTGAGAACCCT
Original  1741 GGAGTATGGCGACATACTGACAACGCAGATTCTTTAGAGCTTACTGTGATCGAAAACCCT
Optimized 1801 GATCACAATTCTTTCTTGGATGAAGAGACTATTTTGGAAGATTTGAAGGTTATGAGAGTT
Original  1801 GATCATAACTCTTTCCTGGATGAAGAGACTATTCTAGAGGATCTTAAGGTCATGCGAGTT
Optimized 1861 ATGTCTAAGAATTACGTTAAAGTTTCTTCTGGTACTACTTTGAGAGAAGCTAGAAACATC
Original  1861 ATGTCAAAGAACTACGTAAAAGTATCCAGCGGAACCACACTCCGCGAAGCGCGCAACATA
Optimized 1921 TTGAAGGAGTCTCATCAAAACTGTATCATGGTTGTTGATGATGATGATTTCTTGGCTGGT
Original  1921 CTTAAAGAATCACACCAGAATTGCATTATGGTCGTTGACGATGATGATTTCTTAGCGGGG
Optimized 1981 ATTTTGACTCACGGAGATATCAGAAGATATTTGTCTAACAACGCTTCTACTATCTTGGAT
Original  1981 ATTCTAACTCACGGAGACATCAGGAGATACCTGAGCAACAACGCTTCCACGATACTTGAT
Optimized 2041 GAAAACACTTGTCCAGTTTCTTCTGTTTGTACTAAGAAAATTTCTTACAGAGGTCAAGAG
Original  2041 GAAAATACCTGCCCGGTAAGTTCCGTTTGCACCAAGAAGATATCGTATCGAGGGCAAGAA
Optimized 2101 AGAGGTTTGTTGACTTGTTATCCAGATGCTACTGTTGGTGTTGCTAAGGAATTGATGGAG
Original  2101 CGGGGACTCCTCACTTGTTATCCCGACGCCACGGTAGGTGTTGCGAAGGAATTGATGGAA
Optimized 2161 GCTAGAGGTGTTAAGCAATTGCCTGTTGTTAAGAGAGGTGAAGTTATTCATAAGGGTAAA
Original  2161 GCACGCGGGGTGAAGCAATTGCCCGTTGTTAAAAGAGGCGAGGTCATCCACAAGGGCAAA
Optimized 2221 AGAAGAAAGTTGTTGGGTTTGTTGCACTATGATTCTATTTGGACTTTCTTGAGAGATGAA
Original  2221 CGCAGAAAACTACTTGGCCTTCTGCATTACGATTCTATTTGGACGTTTCTGAGGGATGAG
Optimized 2281 ATGTCTAGAAGAAGATCTATTAACGATAGAAGAAAGGATAAGGAGGTTGGTACTAATGGT
Original  2281 ATGTCGCGCCGCCGATCAATCAATGATAGACGGAAAGATAAGGAAGTCGGAACCAATGGG
Optimized 2341 CAT
Original  2341 CAT
```

Supplementary Fig. 10: **Codon-optimization of *AtCLCf*.** The full-length original and codon optimized sequences of *AtCLCf*. The codon optimized *AtCLCf* was cloned into *pPICZA* vector for further protein expression in yeast (*Pichia pastoris*).

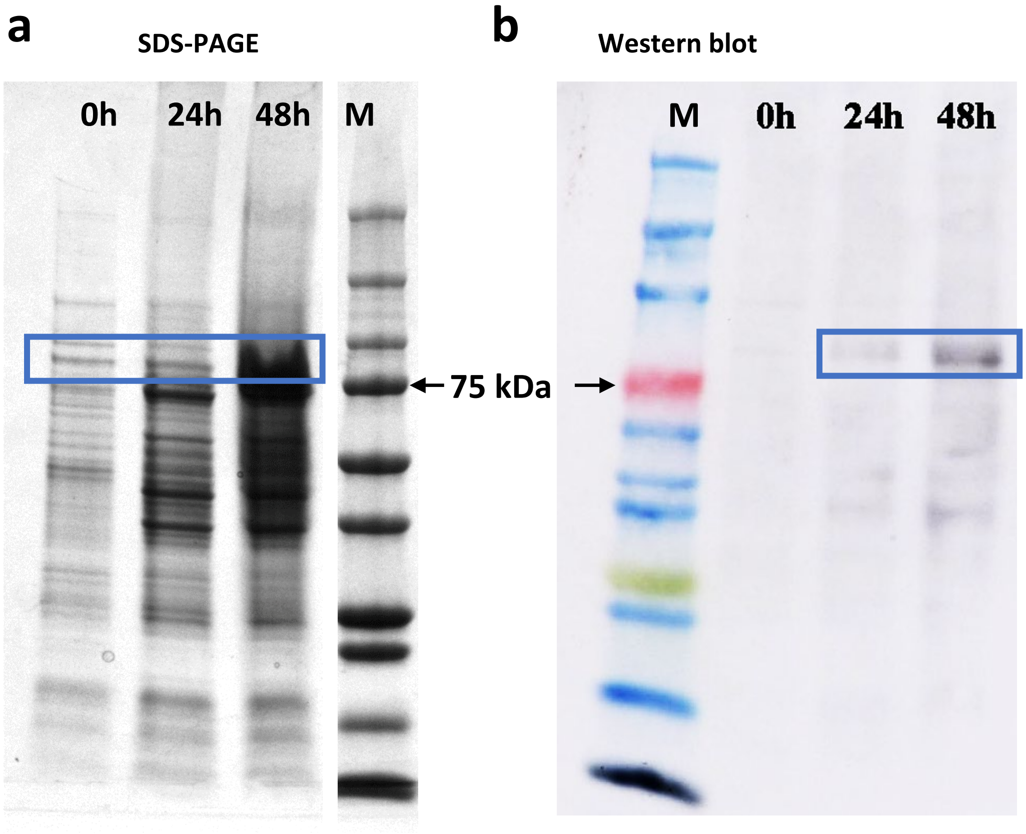

Supplementary Fig. 11: **Recombinant expression of AtCLCf protein.** The AtCLCf protein expression was performed in SMD1168H *Pichia pastoris* strain. Protein expression was induced by adding 5 mM methanol and culture was grown at 28 °C up to 48 h. (**a**) SDS-PAGE shows protein expression of cultures induced from 0 to 48 h. SDS-PAGE was carried out two times. Precision Plus Protein Dual Colour Standards (Bio-Rad) was used as the marker. (**b**) Western blot was performed using the anti-His antibodies to confirm the expression of AtCLCf. The 81 kDa bands, which are of the expected size for AtCLCf, are in the boxed region. Western blot analysis was performed once.

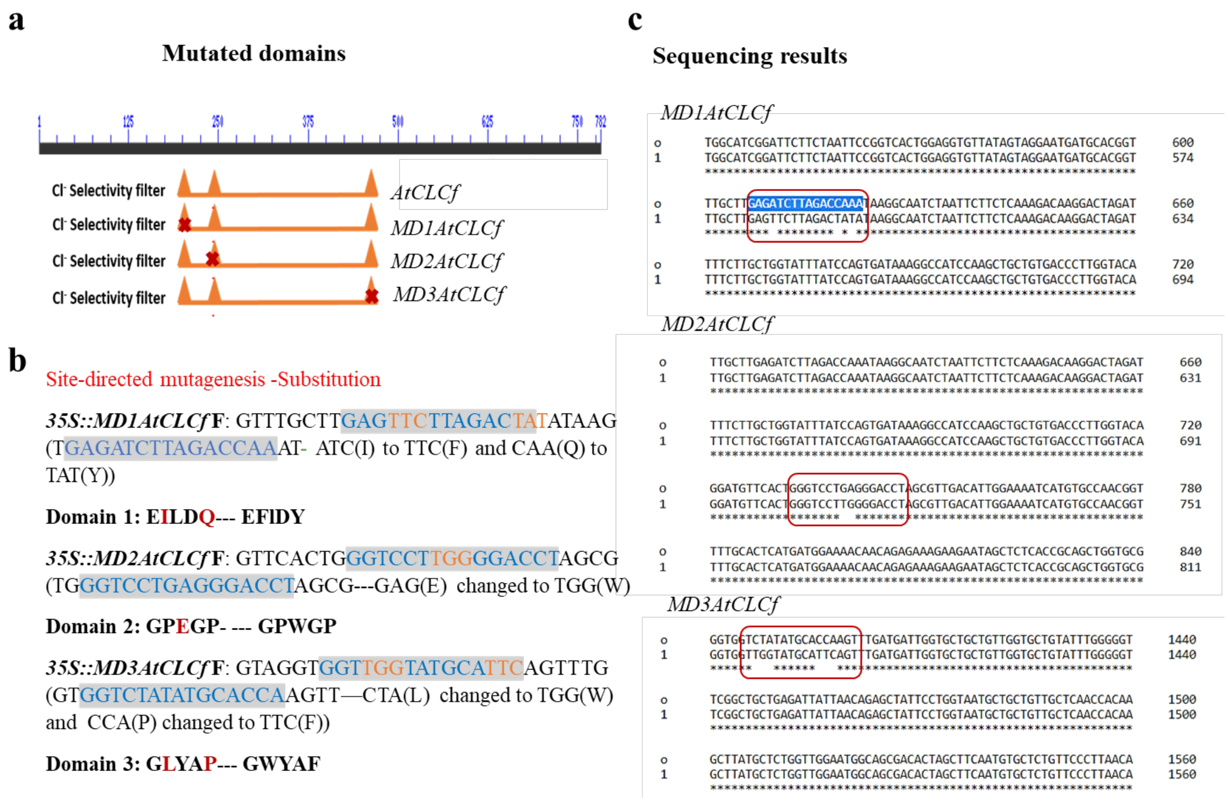

Supplementary Fig. 12: **Details about the mutated sequences in the selectivity filter domain. (a)** Position of three selectivity filter domains and mutated regions in AtCLCf. **(b)** Details of mutated sequences in the three selectivity filter domains. **(c)** Confirmation of mutations by sequencing the mutated regions.

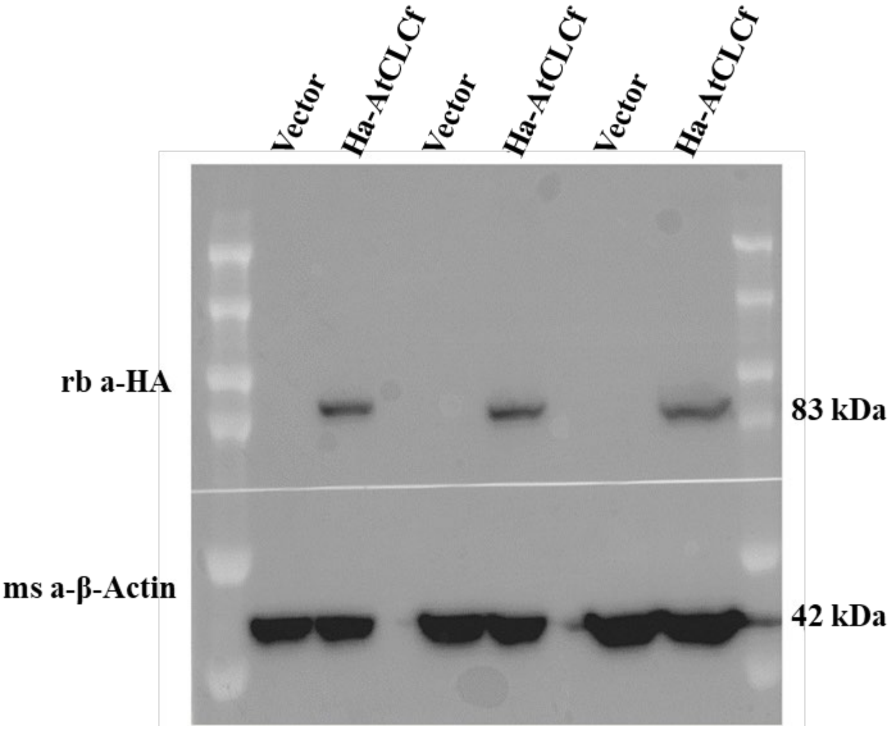

Supplementary Fig. 13: Western blot analysis detected expression of HA-tagged AtCLCf (83 kDa) in Human Embryonic Kidney cell line (HEK293FT cells) expressing HA-AtCLCf, but not in vector transfected cells without AtCLCf, (n = 3). Anti-HA antibodies and anti-β-actin antibodies were used.

Supplementary Table 1: Fresh weights (FW) of the Arabidopsis plants. The data show mean ± SD. Asterisks indicate statistically significant differences (*$P < 0.05$, **$P < 0.01$) between mutant and other genotypes, as measured by unpaired Student's *t*-test (two-tailed), $n = 5$.

Genotype	**Untreated FW (g)**	**Treated FW (g)**
**WT**	2.99 ± 0.37	2.39 ± 0.41*
atclcf	2.45 ± 0.34	1.636 ± 0.28
35S::AtCLCf_T3.1	2.66 ± 0.51	2.392 ± 0.41*
35S::AtCLCf_T3.2	2.56 ± 0.54	2.47 ± 0.22**

Supplementary Table 2: Primers used in this study.

Primers	Forward	Reverse
For cloning *AtCLCf* into pGREEN-OE6HA vector	*AtCLCf*_XhoI CTC GAG ATG TCA TCG GGA GGA GC	*AtCLCf*_SpeI ACT AGT ATG CCC ATT TGT ACC AAC CTC TTT G
**Primers for ChIP-PCR**		
For cloning *pAtClCf::AtCLCf* into GFP vector	GGA TCC TCA CAT CGA GGA AAT TTT GC	ACT AGT ATG CCC ATT TGT ACC AAC CTC
For cloning *pAtClCf* into GUS vector	*GUS*_BamH1: GGA TCC TCA CAT CGA GGA AAT TTT G	*GUS*_Spe1: ACT AGT AAC TGA TTT CAA TTG GCT CTT TT
For yeast complementation assay	*CLCPro*_YEp_EcoR1_: GAATTCTCCTGGGCTTCCT	*CLCPro*_YEp_XmaI: CCCGGGTAGTGTCAAATAATTT
Yeast promoter cloning	*YepPro*_ EcoRI: GAA TTC TCC TGG GCT TCC	*YepPro*_ XmaI: CCC GGG TAG TGT CAA ATA ATT TTA TAG TAT
qRT primers for *AtCLCf*	GTCAAACTCCCAGGAGAGGC	GAAGGGGAACACGAAGCAGA
qRT primers for *AtUBQ10*	CGCCGGCAAGCAGCTAGAGG	ACCACGGAGCCTGAGGACCA
For cloning HA-AtCLCf -pIRES for electrophysiology	EcoRI: AAA AAC GAA TTC ATG TAC CCA TAC GAT GTT CCA GAT TAC GCT ATG TCA TCG GGA GGA GCC	BamH1: AAA AAC GGT ACC TCA AGC GTA ATC TGG AAC ATC GTA TGG GTA ATG CCC ATT TGT ACC AAC
**For mutation of selectivity filter domains**		
MUTD1: *AtCLCf*	GTTTGCTTGAGTTCTTAGACTATATAAG	CTTATATAGTCTAAGAACTCAAGCAAAC
MUTD2:*AtCLCf*	GTTCACTGGGTCCTTGGGGACCTAGCG	CGCTAGGTCCCCAAGGACCCAGTGAAC

MUTD3:*AtCLCf*	GTAGGTGGTTGGTATGCATTCA GTTTG	CAAACTGAATGCATACCAACC ACCTAC

References

1 Santoni, V. Plant plasma membrane protein extraction and solubilization for proteomic analysis. *Methods Mol Biol* **355**, 93-109 (2007). https://doi.org:10.1385/1-59745-227-0:93

2 Palmgren, M. G. *et al.* Sealed inside-out and right-side-out plasma membrane vesicles : optimal conditions for formation and separation. *Plant Physiol* **92**, 871-880 (1990). https://doi.org:10.1104/pp.92.4.871
