# Supplementary material for: The translocation of a chloride channel from the Golgi to the plasma membrane helps plants adapt to salt stress
Source: Nat Commun. 2024 May 10;15:3978. doi: 10.1038/s41467-024-48234-z (PMC11087495; doi:10.1038/s41467-024-48234-z)
Supplement: Supplementary file 3 — Description of Additional Supplementary Files [file 41467_2024_48234_MOESM3_ESM.pdf]

Description of Additional Supplementary Files:

Supplementary Video 1: Salt-induced translocation of AtCLCf to the plasma membrane in Nicotiana benthamiana leaf epidermal cells: Time-lapsed video of sub-cellular translocation of AtCLCf tagged with GFP at the N-terminus to the plasma membrane when treated with 100 mM NaCl for 6h.
