# Supplementary material for: The translocation of a chloride channel from the Golgi to the plasma membrane helps plants adapt to salt stress
Source: Nat Commun. 2024 May 10;15:3978. doi: 10.1038/s41467-024-48234-z (PMC11087495; doi:10.1038/s41467-024-48234-z)
Supplement: Supplementary file 5 — Reporting Summary [file 41467_2024_48234_MOESM5_ESM.pdf]

nature portfolio

Corresponding author(s): Prakash P Kumar

Last updated by author(s): Apr 1, 2024

Reporting Summary

Nature Portfolio wishes to improve the reproducibility of the work that we publish. This form provides structure for consistency and transparency in reporting. For further information on Nature Portfolio policies, see our Editorial Policies and the Editorial Policy Checklist.

Statistics

For all statistical analyses, confirm that the following items are present in the figure legend, table legend, main text, or Methods section.

n/a	Confirmed	
☐	☒	The exact sample size (*n*) for each experimental group/condition, given as a discrete number and unit of measurement
☐	☒	A statement on whether measurements were taken from distinct samples or whether the same sample was measured repeatedly
☐	☒	The statistical test(s) used AND whether they are one- or two-sided<br>*Only common tests should be described solely by name; describe more complex techniques in the Methods section.*
☒	☐	A description of all covariates tested
☒	☐	A description of any assumptions or corrections, such as tests of normality and adjustment for multiple comparisons
☐	☒	A full description of the statistical parameters including central tendency (e.g. means) or other basic estimates (e.g. regression coefficient) AND variation (e.g. standard deviation) or associated estimates of uncertainty (e.g. confidence intervals)
☒	☐	For null hypothesis testing, the test statistic (e.g. *F*, *t*, *r*) with confidence intervals, effect sizes, degrees of freedom and *P* value noted<br>*Give P values as exact values whenever suitable.*
☒	☐	For Bayesian analysis, information on the choice of priors and Markov chain Monte Carlo settings
☒	☐	For hierarchical and complex designs, identification of the appropriate level for tests and full reporting of outcomes
☒	☐	Estimates of effect sizes (e.g. Cohen's *d*, Pearson's *r*), indicating how they were calculated

Our web collection on statistics for biologists contains articles on many of the points above.

Software and code

Policy information about availability of computer code

Data collection	No unpublished software was used. Software built into confocal laser scanning microscope Olympus FV3000, Whole cell recordings were performed with Multiclamp 700b amplifier (Molecular Device).
Data analysis	No unpublished software was used. Image J (https://imagej.nih.gov/ij/download.html), NCBI database, Expasy tool (https://web.expasy.org/translate/) was used to translate the CDS sequences to amino acid sequences and multAlin (http://multalin.toulouse.inra.fr/multalin/) was used to align the amino acid sequences. Phylogenetic analysis was carried out using http://www.phylogeny.fr/. Primers for qRT-PCR were designed using NCBI (https://www.ncbi.nlm.nih.gov/tools/primer-blast/). Transmembrane helices of the proteins were predicted using TMHMM (http://www.cbs.dtu.dk/services/TMHMM/), T-DNA primer design tool (http://signal.salk.edu/tdnaprimers.2.html), Microsoft Excel, PyMOL software (https://pymol.org/2/), StepOneTM Software (v2.1, ABI) for qRT-PCR, Clampfit, and Graphpad Prism 10

For manuscripts utilizing custom algorithms or software that are central to the research but not yet described in published literature, software must be made available to editors and reviewers. We strongly encourage code deposition in a community repository (e.g. GitHub). See the Nature Portfolio guidelines for submitting code & software for further information.

Data

Policy information about availability of data

All manuscripts must include a data availability statement. This statement should provide the following information, where applicable:

- Accession codes, unique identifiers, or web links for publicly available datasets
- A description of any restrictions on data availability
- For clinical datasets or third party data, please ensure that the statement adheres to our policy

Correspondence and material requests should be addressed to Prakash P. Kumar

Field-specific reporting

Please select the one below that is the best fit for your research. If you are not sure, read the appropriate sections before making your selection.

- [x] Life sciences
- [ ] Behavioural & social sciences
- [ ] Ecological, evolutionary & environmental sciences

For a reference copy of the document with all sections, see nature.com/documents/nr-reporting-summary-flat.pdf

Life sciences study design

All studies must disclose on these points even when the disclosure is negative.

Sample size	We conducted all experiments with commonly used sample sizes when the experiments were performed. No prior calculations were performed to determine sample sizes before the experiments.
Data exclusions	No data were excluded from the analyses.
Replication	Experiments were replicated as stated in the figure legends.
Randomization	In all experiments random sampling was done.
Blinding	Blinding was not conducted during data collection and analysis.

Reporting for specific materials, systems and methods

We require information from authors about some types of materials, experimental systems and methods used in many studies. Here, indicate whether each material, system or method listed is relevant to your study. If you are not sure if a list item applies to your research, read the appropriate section before selecting a response.

Materials & experimental systems

n/a	Involved in the study	
[ ]	[x]	Antibodies
[ ]	[x]	Eukaryotic cell lines
[x]	[ ]	Palaeontology and archaeology
[x]	[ ]	Animals and other organisms
[x]	[ ]	Human research participants
[x]	[ ]	Clinical data
[x]	[ ]	Dual use research of concern

Methods

n/a	Involved in the study	
[x]	[ ]	ChIP-seq
[x]	[ ]	Flow cytometry
[x]	[ ]	MRI-based neuroimaging

Antibodies

Antibodies used	All the details are provided in the methods section (including catalog numbers).
Validation	na - we used commercial antibodies for Western blot analyses.

Eukaryotic cell lines

Policy information about cell lines

Cell line source(s)	Cell line used for patch-clamp recordings: Cellosaurus Human Embryonic Kidney 293 (HEK293FT) - Cellosaurus catalog number RRID:CVCL_6911.
Authentication	na
Mycoplasma contamination	na
Commonly misidentified lines (See ICLAC register)	na
